# Development of Multiscale Biological Image Data Analysis: Review of 2006 International Workshop on Multiscale Biological Imaging, Data Mining and Informatics, Santa Barbara, USA (BII06)

**DOI:** 10.1186/1471-2121-8-S1-S1

**Published:** 2007-07-10

**Authors:** Manfred Auer, Hanchuan Peng, Ambuj Singh

**Affiliations:** 1 Life Sciences Division, Lawrence Berkeley National Laboratory, Berkeley, CA, USA; 2 Janelia Farm Research Campus, Howard Hughes Medical Institute, Ashburn, VA, USA; 3 Department of Computer Science, University of California, Santa Barbara, CA, USA

## Abstract

The 2006 International Workshop on Multiscale Biological Imaging, Data Mining and Informatics was held at Santa Barbara, on Sept 7–8, 2006. Based on the presentations at the workshop, we selected and compiled this collection of research articles related to novel algorithms and enabling techniques for bio- and biomedical image analysis, mining, visualization, and biology applications.

## Introduction

With the development of advanced imaging techniques, the number of biological images (e.g. cellular and molecular images, as well as medical images) acquired in digital forms is growing rapidly. Large-scale bioimage databases are becoming available. Analyzing these images has been proven critical for biologists to seek answers to many biological problems. Novel techniques that enable millimeter-, micrometer- and nanometer-scale observations of the same specimen are also emerging. The potential of mining the information in bioimages, especially at different scales of resolution and complexity, is enormous for a deeper understanding of physiology and pathogenesis, for basic sciences as well as for applied sciences and bioengineering. We organized the 2006 International Workshop on Multiscale Biological Imaging, Data Mining and Informatics (BII06) at Santa Barbara, CA, USA, on Sept 7–8, 2006 [1]. It was a follow-up event of the 2005 International Workshop on Bioimage Informatics held at Stanford University [2].

BII06 succeeded in bringing together interdisciplinary researchers to identify problems at each level of imaging and particularly across different imaging modalities/scales, and present their answers using cutting edge image data analysis, computer vision, data mining, machine learning, visualization, and informatics methods. Over 90 people, including 30 faculty members, more than 30 postdoctoral scholars and graduate students, and other scientists from various research institutes, attended the workshop. There were 13 invited talks, 16 peer-reviewed talks, and 14 peer-reviewed posters. The program concluded with a panel discussion that allowed interdisciplinary experts to brainstorm the challenges for effective mining of the increasingly complex bioimage data. All sessions were very interactive. There were a number of questions from the audience and the discussions spilled over into coffee and meal breaks. Short abstracts for the invited talks and two-page papers for all peer-reviewed talks and posters were published in a printed proceedings, which is freely available on the workshop website. Besides the research talks, posters, and the panel, four vendors had product exhibitions at the workshop. Three of them delivered short oral presentations during the lunch hour.

## Challenges of bioimage informatics

Besides reporting a number of exciting bioimaging and image informatics projects, the workshop attendees had an extensive discussion of the following challenges.

### • The demand for bioimage informatics techniques

To biologists, a way to organize and share the large amount of images and search them using metadata or image features is very important. Biologists were of the opinion that image registration and mosaicing are very important image processing tasks. Modeling of processes, at different levels and different resolutions, in order to classify and predict different biological entities and processes is absolutely critical. High-resolution displays provide an opportunity for interactive exploration of data; however, browsing through multiple monitors can be challenging.

### • The need of multiscale imaging

The data from even a single image is huge and we have not yet been able to extract all the information from it. The current deluge of images only exacerbates the challenges. Given the constraints of time and money, is it really worthwhile to spend energy on obtaining multiscale images? Similar questions were raised about high-throughput imaging – is it achievable, is it desirable, is automation the answer? A number of people were of the opinion that as much imaging information should be collected as possible, even though this information cannot be analyzed in depth at this point.

### • Collaboration and communication between biologists and engineers

While biologists and engineers have been using many similar terminologies with distinct meanings (such as "labeling"), it seems that the collaboration and communication between different fields are not going to happen naturally and that it is critical to force the mixture. Both groups of scientists would benefit from this. Tweaking with a microscope for 5 minutes could save tweaking the parameters of a computer algorithm for a few months. Further, data sharing and knowledge sharing should be on a common platform – programs written by computer scientists to be used by biologists should be user-friendly and data provided by biologists should be as complete as possible.

### • Common bioimage informatics problems and bench test data sets

For the image processing and analysis community, *four specific problems *were identified as representative: (1) segmentation, (2) connections in space or time, (3) registration or atlas building, and (4) classification. There is a need to provide a small set of biological datasets with ground truth and a small set of image processing tools that anybody can use and that provides a benchmark for any new algorithms.

### • Modeling

The goal of science is to make *realistic models *of what is happening in nature and often one very important component of realizing those models is biological intuition. There is a need to deal with all the data that can be acquired. The hope is to catalog problems and solutions such that after 15–20 years, the ad-hoc pieces will be integrated together and science can progress. Models should be neither too complex, nor too trivial, to advance the understanding of biology. This raises some fundamental questions, e.g. what to abstract, how to abstract, and how much to abstract.

## Selected papers

We solicited full-paper submissions from the workshop participants who had talk presentations. Each submission was peer-reviewed by at least two reviewers. We accepted 9 papers and compiled this supplement of BMC Cell Biology, an online open access journal. We hope the free-availability of these papers can maximize their visibility.

The contents of these papers include new image analysis and mining algorithms, data visualization, biological applications, enabling supercomputing techniques, and computer vision and machine learning methods to solve other biology problems. In summary, Maree *et al *[3] developed a cell image classification method based on random subwindows and random trees. Long *et al *[4] presented a phenotype clustering analysis for breast epithelial cells, based on the 3D nuclear protein distributions. Altinok *et al *[5] presented a method to extract dynamics information from time-lapse live cell microtubule images. Cecchi *et al *[6] developed a method to extract correlation relationships from brain functional MRI data where there are tens of thousands of variables. Singh [7] presented a new molecule retrieval method based on the similarity of molecule surface information such as shape, field strength and superposition. Peng *et al *[8] developed a suite of techniques to analyze the *in situ *gene expression patterns of fly embryogenesis, and have applied their methods to detecting regulatory motifs of gene sequences and automation of gene expression pattern annotation using anatomical ontology vocabularies. Boucheron *et al *[9] conducted a comparative analysis of both the multispectral and RGB histopathology images, and found a minimal improvement of class prediction accuracy by simply increasing the spectral bands of imaging. Rao *et al *[10] presents a high-performance computing solution to handle image data sets at the gigabyte level, by decomposing 3D image as small segments that are assigned to unique processors of the 3D torus architecture of the IBM Blue Gene/L machine. Staadt *et al *[11] summarized their work on interactive processing and visualization of image data for protein surface, retinal optical coherence tomographic data, and gene expression images of early stage fly embryogenesis.
